# Metal–Organic Framework-Based Chemo-Photothermal Combinational System for Precise, Rapid, and Efficient Antibacterial Therapeutics

**DOI:** 10.3390/pharmaceutics11090463

**Published:** 2019-09-06

**Authors:** Biyuan Wu, Jintao Fu, Yixian Zhou, Yin Shi, Jing Wang, Xiaoqian Feng, Yiting Zhao, Guiling Zhou, Chao Lu, Guilan Quan, Xin Pan, Chuanbin Wu

**Affiliations:** 1 School of Pharmaceutical Sciences, Sun Yat-sen University, Guangzhou 510006, China; 2 College of Pharmacy, Jinan University, Guangzhou 510632, China

**Keywords:** metal–organic frameworks, indocyanine green, anti-bacteria, chemo-photothermal combination therapy

## Abstract

Rapid increase of antimicrobial resistance has become an urgent threat to global public health. In this research, since photothermal therapy is a potential antibacterial strategy, which is less likely to cause resistance, a metal–organic framework-based chemo-photothermal combinational system was constructed. Zeolitic imidazolate frameworks-8 (ZIF-8), a porous carrier with unique features such as high loading and pH-sensitive degradation, was synthesized, and then encapsulated photothermal agent indocyanine green (ICG). First, ICG with improved stability in ZIF-8 (ZIF-8-ICG) can effectively produce heat in response to NIR laser irradiation for precise, rapid, and efficient photothermal bacterial ablation. Meanwhile, Zn^2+^ ions released from ZIF-8 can inhibit bacterial growth by increasing the permeability of bacterial cell membrane and further strengthen photothermal therapy efficacy by reducing the heat resistance of bacteria. Study showed that bacteria suffered from significant changes in morphology after treatment with ZIF-8-ICG under laser irradiation. The combinational chemo-hyperthermia therapy of ZIF-8-ICG could thoroughly ablate murine subcutaneous abscess induced by methicillin-resistant *Staphylococcus aureus* (MRSA), exhibiting a nearly 100% bactericidal ratio. Both in vitro and in vivo safety evaluation confirmed that ZIF-8-ICG was low toxic. Overall, our researches demonstrated that ZIF-8-ICG has great potential to be served as an alternative to antibiotics in combating multidrug-resistant bacterial pathogens.

## 1. Introduction

The widely spread bacterial pathogens used to be a serious threat to human health due to the lack of specific medicine and therapeutic measure. Before the discovery of antibiotics, people can only affect the development of the disease by conservative approaches, such as isolating infectious patients, which led to the extremely low survival rate [1,2]. Over the past decades, remarkable progress has been achieved in developing antibiotics for bacterial infection. However, the return to the pre-antibiotic era has become a reality in many parts of the world because of the rapid emergence of multidrug-resistant (MDR) bacterial pathogens, such as “ESKAPE” pathogens (*Enterococcus faecium*, *Staphylococcus aureus*, *Klebsiella pneumonia*, *Acinetobacter baumannii*, *Pseudomonas aeruginosa* (*P. aeruginosa*) and *Enterobacter species*) [3,4,5]. Recent researches showed that failing to manage MDR bacterial infections may cause more than 10 million deaths per year and cost up to 100 trillion dollars by 2050 [6,7]. In this situation, it is quite imperative to develop alternative therapeutic strategies for bacterial infection with minimizing resistance emergence.

Heating is recognized as a classic and effective sterilization method, which is less likely to result in drug resistance and widely used in families, food, medical, and industrial production [8]. However, the difficulty of heating in achieving precise and accurate treatment has hampered its clinical applications. Recently, photothermal therapy has attracted increasing attention in the treatment of bacterial infection [9,10,11,12,13]. It utilizes photothermal agents to convert near-infrared (NIR) light energy into heat with minimal thermal damage to healthy tissue. The increasing local temperature can further damage bacterial cell membrane, denature enzyme, and eventually cause the death of bacteria. Up to now, though more and more photothermal agents have been developed, only indocyanine green (ICG) is approved by the United States Food and Drug Administration (FDA) for clinical applications [14,15]. However, the administration of free ICG is still limited by its intrinsic physicochemical properties, such as poor photostability and thermal stability and rapid aggregation and degradation in polar solvents [16,17]. To overcome these limitations, various efforts have been focused on developing an ideal nanocarrier for ICG encapsulation [18,19], which was desired to develop a simple and facile preparation process, encapsulate cargos with high drug loading capacity, and stabilize ICG from degradation.

A candidate carrier to meet these challenges may lie in the recently developed nanoporous materials known as metal–organic frameworks (MOFs). They are an emerging class of hybrid porous materials based on coordination bonds between metal ions (or clusters) and organic ligands [20]. With the unique properties, including extremely large surface area and ordered porous interior, MOFs can be employed as promising candidates to encapsulate a large amount of drug molecules. Their spatial confinement of porous structure is beneficial to stabilizing guest molecules, restricting molecules movement, and subsequently avoiding aggregation [21,22]. During the degradation process of MOFs, metal ions such as Ag^+^ and Zn^2+^ ions can be liberated and further contribute to damaging bacteria by directly disrupting bacterial membrane and intracellular proteins for chemotherapy [23].

**Scheme 1:**
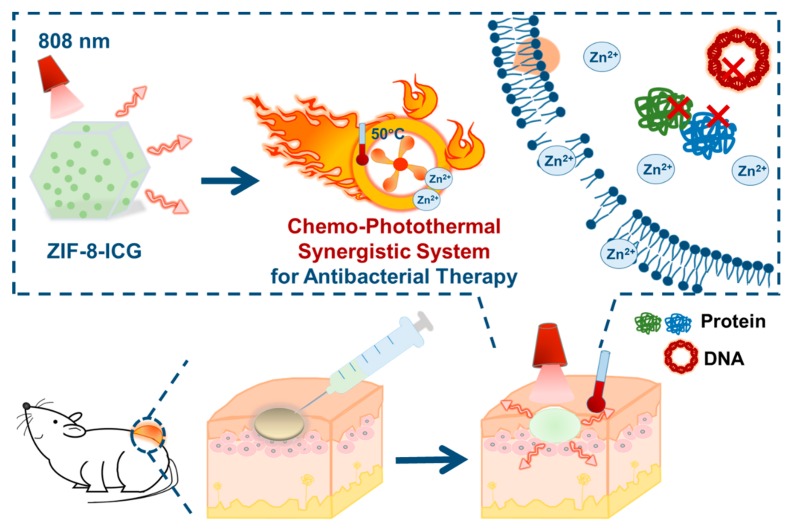
Schematic illustration of the chemo-photothermal combinational nanosystem for the treatment of methicillin-resistant *Staphylococcus aureus* (MRSA) infection.

In this research, zeolitic imidazolate frameworks-8 (ZIF-8), a representative subclass of MOFs, is expected to be an ideal nanocarrier for effective chemo-photothermal combination therapy (Scheme 1). First, ZIF-8 can be easily prepared by the reaction of Zn^2+^ ions and 2-methylimidazole at room temperature by mild stirring. Besides, the unique porous structure of ZIF-8 can encapsulate sufficient ICG to form ICG-loaded ZIF-8 nanoparticles (ZIF-8-ICG) for photothermal bacterial ablation. Meanwhile, since ZIF-8 shows great stability in physiological environment but gradually degrades at acidic medium, Zn^2+^ ions can be released from ZIF-8 in infectious site (pH < 6.5), which is beneficial to inhibiting bacterial growth and further improving the antibacterial efficacy of photothermal therapy by reducing their heat resistance [24,25,26]. The successful fabrication of ZIF-8-ICG was verified by scanning electron microscopy (SEM), powder X-ray diffraction (PXRD), Fourier transform infrared spectroscopy (FT-IR), and thermal gravimetric analysis (TGA), and its drug loading capacity was determined to be 26.3%. Standard agar plate method and optical density measurement confirmed the high in vitro antibacterial activity of ZIF-8-ICG. To further evaluate the in vivo activity, the skin of mice was infected by methicillin-resistant *Staphylococcus aureus* (MRSA) and treated with ZIF-8-ICG via chemo-photothermal combination therapy. We expected to demonstrate that ZIF-8-ICG was a nanosystem combining the chemo-photothermal therapy together, which possessed efficient localized chemo-photothermal bacterial ablation and disinfection activities and was recognized as an effective weapon in combating the growing threat of MDR bacteria.

## 2. Materials and Methods

### 2.1. Materials

Zinc nitrate hexahydrate was obtained from Meryer Chemical Technology Co., Ltd. (Shanghai, China). 2-methyl imidazole and ICG were purchased from Aladdin Bio-Chem Technology Co., Ltd. (Shanghai, China). Fetal calf serum (FBS) and Dulbecco’s modified eagle medium (DMEM) were provided by Gibco Life Technologies (Grand Island, NY, USA). CCK-8 was acquired from Dojindo Laboratories (Kumamoto Prefecture, Japan). Methanol (AR, >99.7%) was obtained from Tianjin Kemiou Chemical Reagent Co., Ltd. (Tianjin, China). All the reagents were used as received without further purification.

### 2.2. Synthesis of ZIF-8-ICG

The ICG-loaded ZIF-8 was prepared by a facile one-pot method at room temperature. For the typical synthesis, 30 mg of zinc nitrate hexahydrate and a defined amount of ICG were dissolved into 2 mL of methanol. Subsequently, 66 mg of 2-methyl imidazole was added into the above uniform solution and the resulting mixture was constantly stirred for 1 h. The green precipitate was then collected by centrifugation at 10,000 rpm for 10 min and further washed three times with methanol to get rid of the unreacted reactants. Finally, the obtained green product was dried at room temperature in a vacuum oven.

### 2.3. Characterization

The morphology of ZIF-8-ICG was observed by SEM (JSM-6330F, Japanese Electronics Co., LTD, Akashima, Japan). The samples were placed on a brass stub and then sputter-coated with gold for two cycles before examination. The N_2_ absorption–desorption isotherm of the sample as well as pore size distribution were measured using automatic volumetric sorption analyzer (ASAP2460, Micromeritics Instrument Corp, Atlant, GA, USA). The crystalline structure was evaluated by X-ray powder diffraction (PXRD, D8 Advance, Bruker, Karlsruhe, Germany) using Cu Kα radiation with 2*θ* in the range of 3°–40° at a scanning rate of 5° min^−1^. The Fourier infrared analysis was carried out by Fourier transform infrared spectrometry (STA449F3/Nicolet6700, Thermo Electron Corporation, Waltham, MA, USA). Thermal gravimetric analysis (TGA) measurement was performed by Thermogravimetry (TG209F1 libra, Netzsch, Selb, Germany).

### 2.4. Photothermal Effect of ZIF-8-ICG

One milliliter of ZIF-8-ICG aqueous suspension (1 mg·mL^−1^) was added into the 1.5 mL Eppendorf tube and exposed to 808 nm near-infrared laser (1 W·cm^−2^) for 30 min. The temperature was recorded by an infrared thermal sensing and imaging equipment (TiS75, Fluke, Everett, WA, USA) every 5 min.

### 2.5. In Vitro Hyperthermia Antibacterial Activity

MRSA and *P. aeruginosa* were incubated with Luria-Bertani (LB) medium overnight. The bacteria in the logarithmic phase was diluted to 2 × 10^5^ colony forming units (CFU)·mL^−1^. Qualified amount of ZIF-8-ICG was suspended by sterilized PBS solution and diluted to a broad concentration range from 1000 µg·mL^−1^ to 31.3 µg·mL^−1^. Then the pretreated bacterial suspension was incubated with ZIF-8-ICG at room temperature for 2 h, followed by irradiation under NIR laser (1 W·cm^−2^) for 30 min. The increased temperature was recorded by an infrared thermal sensing and imaging equipment. Then the bacterial suspension was serially 10-fold diluted, and subsequently 10 μL of each diluted sample was spotted on agar plates. The plates were then incubated at 37 °C overnight. Finally, the in vitro hyperthermia antibacterial activity was evaluated by the number of grown colonies.

### 2.6. Growth Kinetics Curve of Bacteria with Different Treatments

MRSA and *P. aeruginosa* in the logarithmic phase were diluted to 2 × 10^5^ CFU·mL^−1^ with LB culture medium. Then 50 µL of bacterial suspension was seeded into the 96-well plate followed by addition of 50 µL of nanoparticle suspension (1000.0 µg·mL^−1^) to the wells. After incubation at room temperature for 2 h, laser irradiation was conducted to some groups for 30 min. Then the 96-well plate was incubated at 37 °C for further observation. The optical density of the bacterial suspension at 630 nm was tested and recorded by a microplate reader at predetermined time intervals.

### 2.7. Morphology Investigation of Bacteria

MRSA and *P. aeruginosa* in the logarithmic phase were collected. Bacteria without treatment (control) and with various concentrations of ZIF-8-ICG treatments followed by 30 min NIR laser irradiation were washed three times by PBS solution and collected by centrifugation (3000 rpm, 15 min). Then the bacteria were quickly fixed with 2.5% glutaraldehyde overnight at 4 °C. After that, the bacteria were dehydrated through sequential treatments of 30%, 50%, 70%, 80%, 90%, 95%, and 100% ethanol solutions for 15 min. The morphology of the bacteria was observed by SEM.

### 2.8. Cytotoxicity Study

In order to investigate the biocompatibility of ICG-loaded nanoparticles, the in vitro cytotoxicity was performed in 293T cell lines using the CCK-8 viability assay and the cells were obtained from Chinese Academy of Sciences Cell Bank (Shanghai, China). Cells were seeded in the 96-well plate and incubated in 5% CO_2_ at 37 °C. After incubation for 24 h, the medium was replaced by 100 µL of pure medium (blank control) and fresh medium containing different concentrations of ZIF-8-ICG. After further incubation with the samples for 24 h, 10 µL of CCK-8 solution was added into the wells. The absorbance was measured at 450 nm by a microplate reader. The relative viability was calculated by comparing the treated cells with blank control and relevant data were expressed as mean ± standard deviation (SD) of six independent wells.

### 2.9. In Vivo Antibacterial Assessment and Safety Evaluation

Five-week-old Balb/c mice were selected as the skin-infected model animals. The back hair (approximately 2 × 2 cm^2^) was shaved off one day before modeling. The bacterial inoculum was prepared by washing the logarithmic phase MRSA three times with PBS solution and then diluted to 1 × 10^8^ CFU·mL^−1^. The pretreated mice were subcutaneously injected with 100 µL of MRSA inoculum and allowed to infect for 24 h. Then the infected mice were randomly divided into six groups for further treatments (five mice in each group), including PBS, PBS+NIR, ZIF-8, ZIF-ICG, ZIF-8-ICG-L+NIR (low dose group, 1 mg·mL^−1^), and ZIF-8-ICG-H+NIR (high dose group, 10 mg·mL^−1^). Exposure to laser irradiation for 30 min (1 W·cm^−2^) was further conducted 2 h after administration in PBS+NIR, ZIF-8-ICG-L+NIR, and ZIF-8-ICG-H+NIR groups. Following different treatments, the mice were sacrificed and the infected skin tissues were photographed, harvested, and homogenized. The homogenized suspensions were serially 10-fold diluted, and subsequently 10 μL of each diluted sample was spotted on agar plates. After incubation at 37 °C overnight, the number of bacterial colonies was counted to evaluate the in vivo antibacterial activity. Moreover, the major organs including heart, liver, spleen, lung, and kidney were collected for histological assessment. All animal experiments in this research were supervised and approved by the Animal Ethics Committee of Shantou University Medical College (SUMC2019-348, 5 March 2019).

## 3. Results

### 3.1. Synthesis and Characterization of ZIF-8-ICG

**Figure 1:**
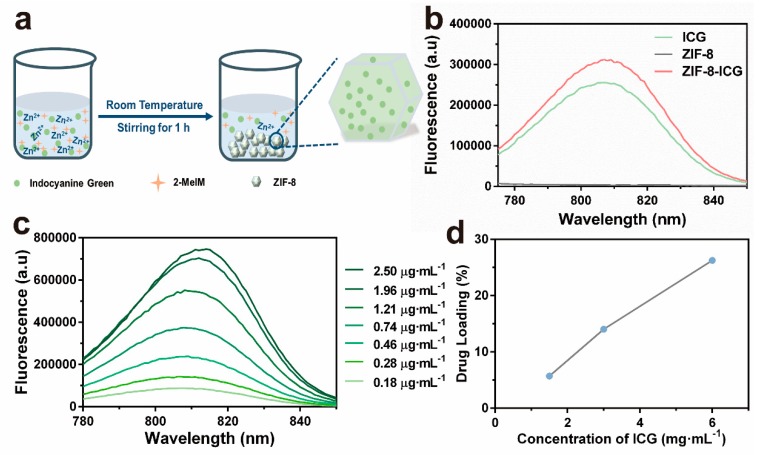
(**a**) Schematic representations for the in situ fabrication process of indocyanine green (ICG)-loaded zeolitic imidazolate frameworks-8 (ZIF-8) nanoparticles (ZIF-8-ICG); (**b**) The fluorescent spectrum of ICG, ZIF-8, and ZIF-8-ICG; (**c**) Fluorescent spectra of ICG solution with different concentrations at the 760 nm excitation wavelength; (**d**) The ICG-loading capacity of ZIF-8 by optimizing the reactant concentration of ICG.

ICG-loaded ZIF-8 was fabricated by a facile, green, one-pot method according to the previous literature [16]. Briefly, ZIF-8-ICG was obtained by stirring the mixture of zinc nitrate hexahydrate, 2-methyl imidazole, and ICG at room temperature for 1 h (Figure 1a). Fluorescent spectrum was used to verify the successful encapsulation of ICG into ZIF-8. As shown in Figure 1b, ZIF-8-ICG suspension showed the characteristic fluorescence peak of ICG while no absorption was observed in pure ZIF-8 nanoparticles. Hence, fluorescent spectrum was selected as the quantitative method of ICG (Figure 1c). In order to initiate efficient photothermal effect, the concentration of ICG during one-pot reaction was optimized. As shown in Figure 1d, the loading capacity of ICG increased with the increase of ICG concentration in the reactant solution. Thus, the optimal ICG concentration was selected as 6 mg·mL^−1^ with the corresponding loading capacity of ICG confirmed as 26.3%.

**Figure 2:**
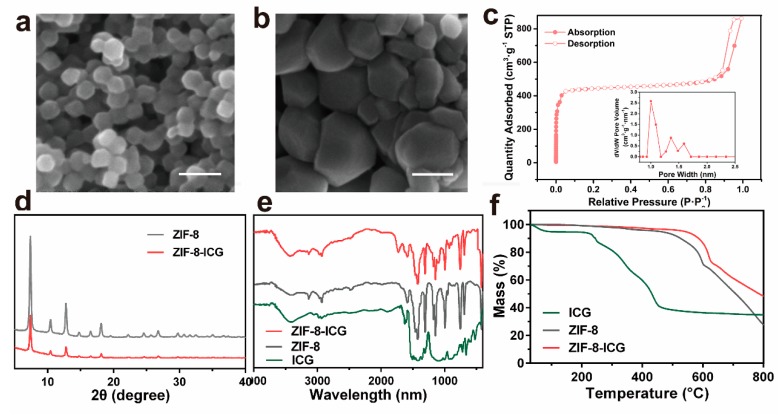
SEM images of (**a**) ZIF-8 and (**b**) ZIF-8-ICG (Scale bar: 100 nm); (**c**) N_2_ adsorption-desorption isotherm of ZIF-8, the insert was pore size distribution of ZIF-8; (**d**) powder X-ray diffraction (PXRD) patterns of ZIF-8 before and after ICG loading; (**e**) FT-IR spectra of ICG, ZIF-8, and ZIF-8-ICG; (**f**) TGA results of ICG, ZIF-8, and ZIF-8-ICG.

The morphology and particle size of nanoparticles were observed by scanning electron microscopy (SEM). Figure 2a,b revealed that the ZIF-8 and ZIF-8-ICG both appeared as typical hexagon morphology, suggesting that the ICG encapsulation would not change the morphology of ZIF-8. In addition, the particle size of ZIF-8-ICG was a bit larger than that of ZIF-8, which might be attributed to the influence of ICG on the process of nucleation and growth of ZIF-8 [27]. Quantitative information about the structure of porous materials can be usually analyzed by N_2_ absorption and desorption measurement. As shown in Figure 2c, the BET surface area and pore size of ZIF-8 were calculated as 1836.4 cm^2^·g^−1^ and 0.8 nm, respectively. The extremely large surface area and porous interiors contributed to encapsulate large amount of ICG molecules into the pores of ZIF-8 and subsequently stabilize the photothermal agent by the spatial confinement effect. The crystallographic structure of ZIF-8-ICG was characterized by powder X-ray diffraction (PXRD). Figure 2d revealed that the ZIF-8-ICG exhibited similar crystalline characteristic peaks to ZIF-8, which demonstrated that the encapsulation of ICG did not damage the natural crystalline structure of ZIF-8. Additionally, the chemical structure of all samples was investigated by Fourier transform infrared spectroscopy (FT-IR). As shown in Figure 2e, the characteristic peaks of ZIF-8 including C–H stretch, aliphatic C–H stretch of the imidazole, and Z–N stretching mode were in good agreement with the published literatures [28,29]. The spectrum of ZIF-8-ICG was mostly similar to that of ZIF-8 with no characteristic bonds of ICG observed, revealing that the encapsulation of ICG mainly resulted from physical absorption. The thermal gravimetric analysis (TGA) result of ZIF-8 and ZIF-8-ICG was exhibited in Figure 2f. Both ZIF-8 and ZIF-8-ICG kept stable even the temperature increased to 400 °C. By contrast, free ICG showed an obvious mass decline at 100 °C which was ascribed to the free water evaporation, followed by a continuous mass decline when the temperature was over 200 °C. These results indicated that the encapsulation of ICG into the pores of ZIF-8 was beneficial to improving the thermal stability of ICG.

### 3.2. In Vitro Photothermal Ability of ZIF-8-ICG

**Figure 3:**
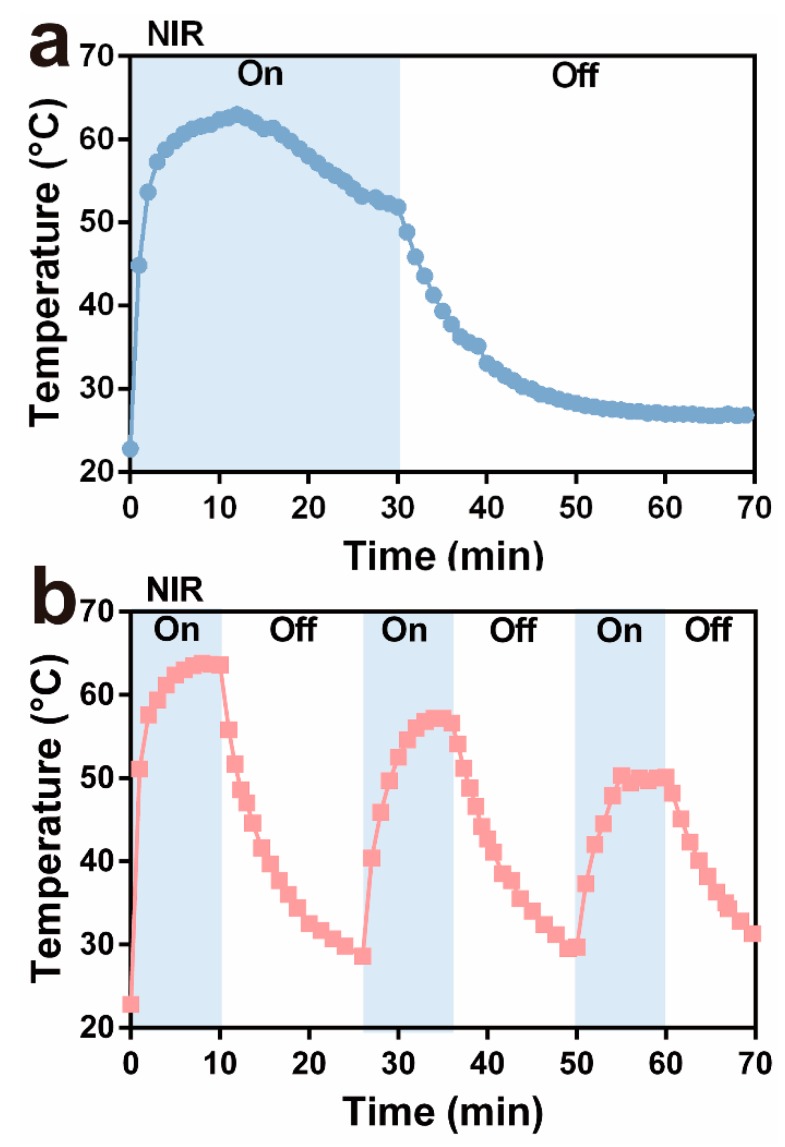
Change in thermal curves of ZIF-8-ICG suspensions after (**a**) one time irradiation and (**b**) three times irradiation for 30 min.

In order to evaluate the photothermal ability of ZIF-8-ICG, the nanoparticle suspension in the Eppendorf tubes was irradiated under 808 nm laser for 30 min, and the real-time temperature was recorded by an infrared thermal sensing and imaging equipment. Moreover, the influence of irradiation pattern was investigated. Briefly, the circulation of irradiation was set as one time (30 min) and three times (10 min for each time, and the next irradiation was conducted when the sample cooled to the ambient temperature). As depicted in Figure 3a, one circulation group showed high temperature over 50 °C for approximately 30 min which was longer than 20 min in the group of three circulations (Figure 3b). As revealed in the previous studies [11,30], increasing temperature over 50 °C could damage bacterial cell membrane, denature enzyme, and eventually cause the death of bacteria. The irradiation model of one circulation presented superior hyperthermia ability than three circulations. Therefore, the following research was conducted by one circulation irradiation pattern for total 30 min.

### 3.3. In Vitro Antibacterial Property of ZIF-8-ICG

**Figure 4:**
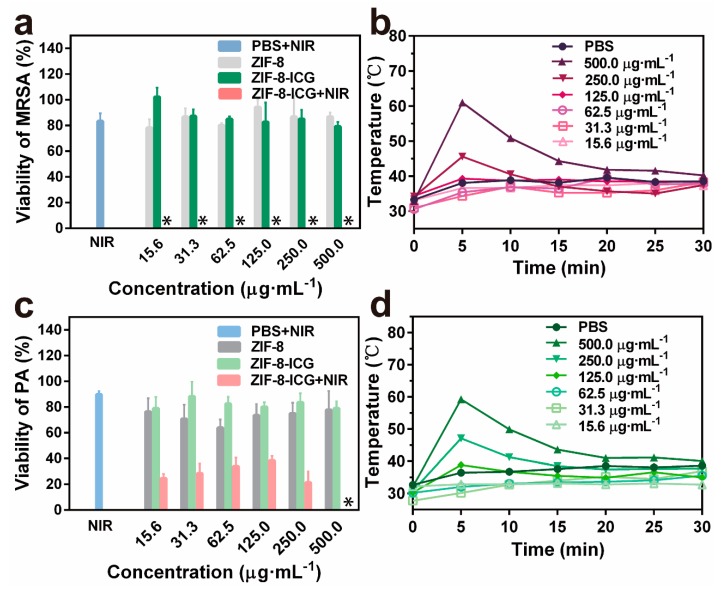
(**a**) The bacterial viability of MRSA treated by different samples with gradient concentrations (∗ denotes no colonies were observed); (**b**) Temperature evolution profile of ZIF-8-ICG suspensions with different concentrations in the presence of MRSA under laser irradiation for 30 min; (**c**) The bacterial viability of *P. aeruginosa* treated by different samples with gradient concentrations; (**d**) Temperature evolution profile of ZIF-8-ICG suspensions with different concentrations in the presence of *P. aeruginosa* under laser irradiation for 30 min.

**Figure A1:**
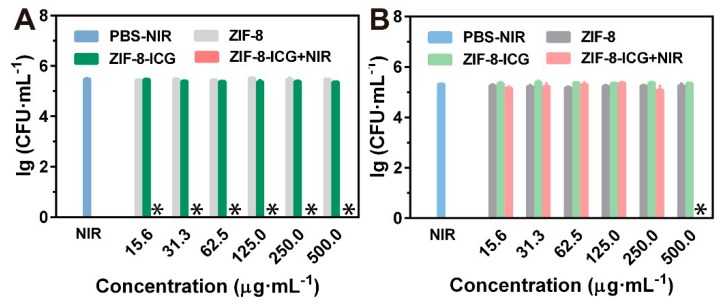
Bacteria viabilities of (**a**) MRSA and (**b**) *P. aeruginosa* with different treatments (∗ denotes no colonies were observed).

To investigate the in vitro chemo-photothermal effect of ZIF-8-ICG, bacteria were cultured with different samples and further irradiated with a laser, using standard agar plate method to evaluate the viability of bacteria. MRSA and *P. aeruginosa* were selected as the representative strains of Gram-positive bacteria and Gram-negative bacteria, respectively. As shown in Figure 4a, ZIF-8-ICG+NIR treated group achieved ~100% MRSA killing efficiencies at a broad concentration range of 15.6~500.0 µg·mL^−1^, while PBS+NIR, ZIF-8, and ZIF-8-ICG treated groups showed no significant influence on the viability of MRSA (Figure 4a). Similarly, the percentage of *P. aeruginosa* survival in ZIF-8-ICG+NIR treated group was lower than 40% (Figure 4c), indicating the efficient bactericidal effect of photothermal therapy. The viabilities of bacteria in logarithmic indication were presented in Figure A1. It should be mentioned that when the concentration of ZIF-8-ICG in bacterial suspension was lower than 250.0 µg·mL^−1^, NIR laser irradiation could not result in great temperature increase (lower than 50 °C), as shown in Figure 4b,d. The outstanding photothermal bactericidal activity of ZIF-8-ICG+NIR treated group at low temperature demonstrated the importance of combining photothermal therapy with chemotherapy, because Zn^2+^ ions released from ZIF-8 increased the permeability of bacterial cell membrane and reduced the heat resistance of bacteria.

**Figure 5:**
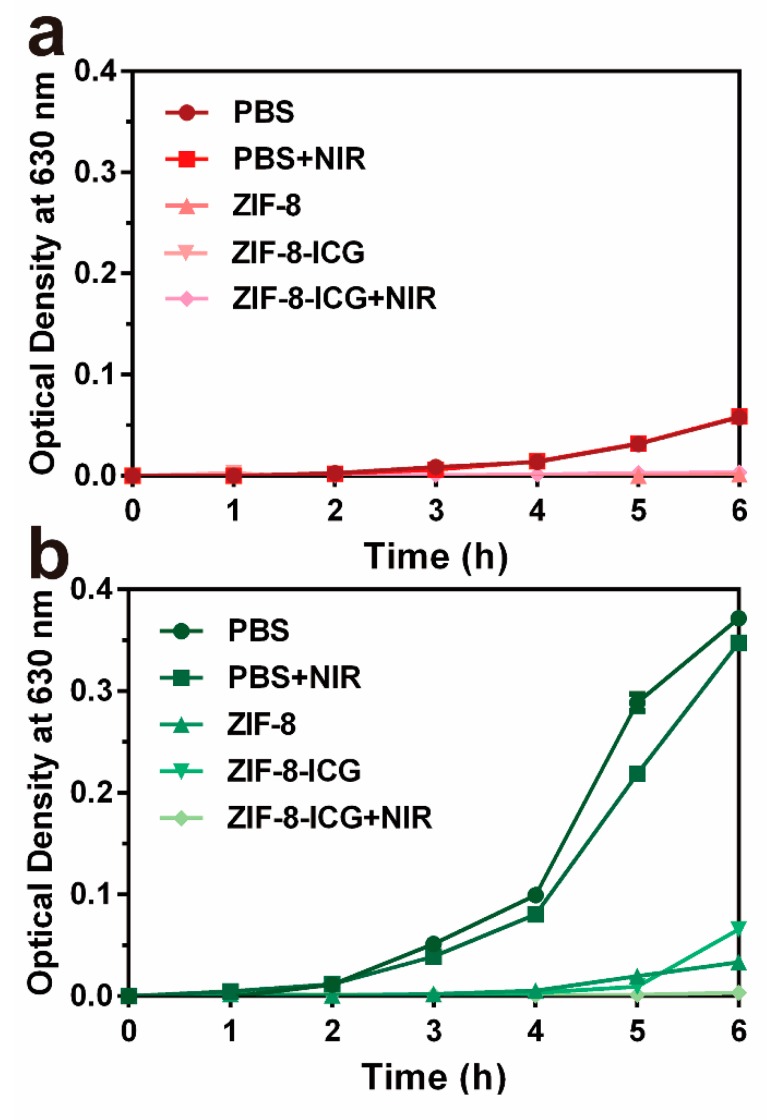
The growth kinetics curve of (**a**) MRSA and (**b**) *P. aeruginosa* with different treatments for 6 h at 37 °C.

Furthermore, the bacterial growth kinetics were investigated by incubating different nanoparticles with MRSA and *P. aeruginosa* at 37 °C, followed by measuring the optical density at predetermined time intervals. As shown in Figure 5a,b, while laser irradiation had negligible influence on inhibiting the growth of MRSA and *P. aeruginosa*, the optical densities increases of both ZIF-8 and ZIF-8-ICG treated groups were obviously restrained. The results indicated the unique advantage of ZIF-8 as a carrier for antimicrobial application. Since the pH of bacterial infected microenvironment is usually lower than 6.5, abundant Zn^2+^ ions can be released from both ZIF-8 and ZIF-8-ICG showing chemotherapeutic effect on inhibiting bacterial growth. Moreover, complete growth inhibition of MRSA and *P. aeruginosa* was observed in the group of ZIF-8-ICG with NIR laser irradiation, which further illustrated the great bactericidal efficacy of photothermal therapy.

**Figure 6:**
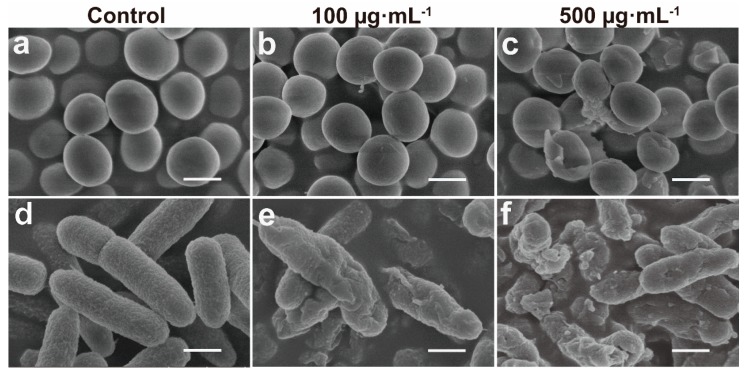
SEM images of (**a**–**c**) MRSA and (**d**–**f**) *P. aeruginosa* after various treatments (Scale bar: 1 µm).

To further understand the mechanism of the antimicrobial functions of the ZIF-8-ICG with NIR laser irradiation, we investigated the morphological changes of bacteria before and after treatment through SEM. As shown in Figure 6a,d, smooth surface was observed in both untreated MRSA and *P. aeruginosa* group. After incubation with ZIF-8-ICG under laser irradiation for 30 min, both MRSA and *P. aeruginosa* cell surface become rougher and more wrinkled with concave holes as the concentration of ZIF-8-ICG was increased (Figure 6b,c,e,f). Obviously distorted cell shape and leakages of cytoplasmic contents could be observed, when cells were treated with ZIF-8-ICG at a concentration higher than 500 µg·mL^−1^. The results illuminated the significant influence of photothermal ablation on disrupting bacterial morphology.

The prominent in vitro photothermal antibacterial ability of ZIF-8-ICG may be attributed to the chemo-photothermal synergistic effect of ZIF-8-ICG instead of the monotherapy of chemo or photothermal. These data suggest that the mechanism of action of ZIF-8-ICG is as follows: (1) ZIF-8 is stable under general physiological condition and delivers ICG efficiently to infected site; (2) Bacteria growth and metabolism causes the reduction of microenvironment pH level, which promotes the pH-sensitive degradation of ZIF-8 inducing Zn^2+^ ions release; (3) Cationic Zn^2+^ ions interact with the anionic bacterial membrane by electrostatic interaction, subsequently inhibit bacteria growth and reduce their heat resistance by resulting in a serious structural deformation of the bacterial cell membrane; (4) NIR laser-triggered photothermal therapy thus exhibits precise, rapid, and efficient antibacterial effect against various bacterial species; (5) The hyperthermia induced by photothermal therapy further triggers more Zn^2+^ ions release, resulting in further improvement on bactericidal efficiency of chemotherapy.

### 3.4. In Vivo Antibacterial Study and Safety Evaluation

**Figure 7:**
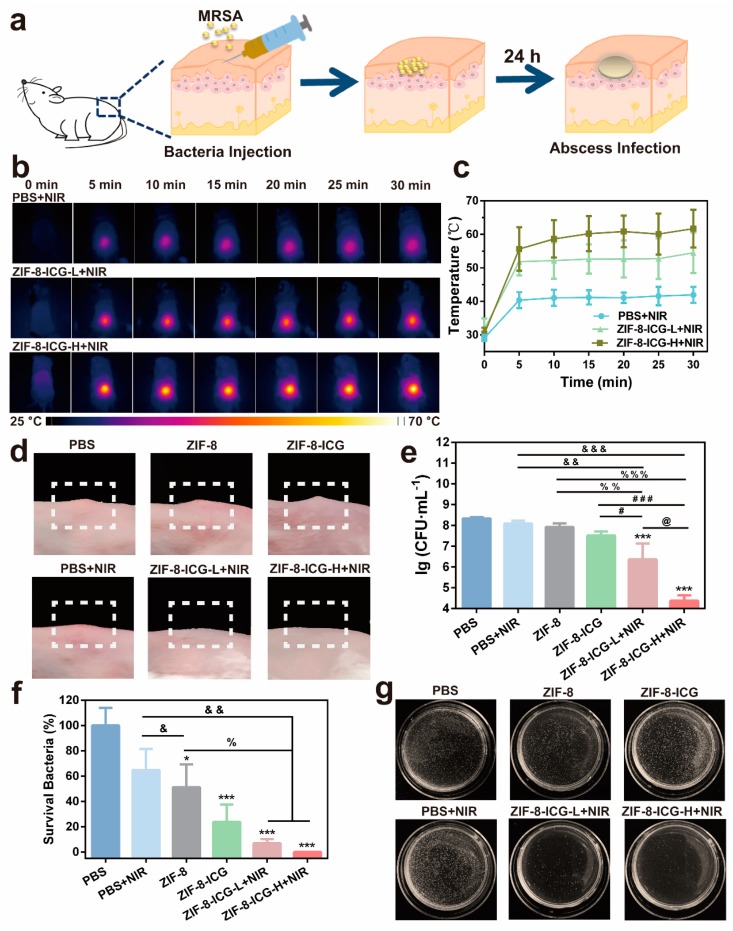
(**a**) Schematic illustration of the mice modeling process by local injection; (**b**) The thermographic images of MRSA-infected mice under 808 nm laser irradiation at 0, 5, 10, 15, 20, 25, and 30 min post-administration; (**c**) Local temperature profiles of laser treated mice at different time points; (**d**) Digital photographs of the infected skin surface after different treatments; (**e**) Microbial burden after different treatments in murine models of skin; (**f**) Bacterial viability of MRSA after different treatments. * *p* < 0.05 vs. PBS; *** *p* < 0.001 vs. PBS; & *p* < 0.05 vs. PBS+NIR; && *p* < 0.01 vs. PBS+NIR; &&& *p* < 0.001 vs. PBS+NIR; % *p* < 0.05 vs. ZIF-8; %% *p* < 0.01 vs. ZIF-8; %%% *p* < 0.001 vs. ZIF-8; # *p* < 0.05 vs. ZIF-8-ICG; ### *p* < 0.001 vs. ZIF-8-ICG; @ *p* < 0.05 vs. ZIF-8-ICG-L+NIR; (**g**) Photographs of MRSA colonies in the agar plates.

To further verify the antibacterial activity, a MRSA-induced murine subcutaneous abscess model was performed [26]. The infected mice with subcutaneous abscesses were divided randomly into six groups and treated with PBS, PBS+NIR, ZIF-8, ZIF-8-ICG, ZIF-8-ICG-L+NIR (low dose, 1 mg·mL^−1^), and ZIF-8-ICG-H+NIR (high dose, 10 mg·mL^−1^) by intralesional injection, respectively (Figure 7a). The skin appearance and local temperature at infected site was recorded during the whole therapeutic period. The therapeutic efficacy was evaluated by comparing the number of bacterial colonies in the infected skin homogenate. Figure 7b,c showed that the local temperature of NIR laser-treated groups increased rapidly in 5 min and well maintained in the following 25 min. Moreover, the local high temperature was concentrated precisely and accurately on the laser irradiation region. Compared with PBS+NIR group, the local temperature of ZIF-8-ICG-L+NIR and ZIF-8-ICG-H+NIR groups could be over 50 °C, and their maximum temperature increased with the increase of ZIF-8-ICG dosage. The hyperthermia effect could not only contribute to denaturing the protein of bacteria but also accelerate the degradation of ZIF-8 to release out free Zn^2+^ ions. After photothermal analysis, the mice were sacrificed and the skin tissues were photographed. As shown in Figure 7d, MRSA infection induced obvious subcutaneous abscesses. The groups of PBS and PBS+NIR showed negligible activity against MRSA infection, while the groups of ZIF-8-ICG-L+NIR and ZIF-8-ICG-H+NIR could realize effective thermal ablation of abscesses without causing obvious burnt or bleeding phenomena. Infected tissues were collected and grinded into homogenate for bacterial colony counting. As shown in Figure 7e,f, the bacteria concentration in the infected tissues of PBS, PBS+NIR, ZIF-8, ZIF-8-ICG, ZIF-8-ICG-L+NIR, and ZIF-8-ICG-H+NIR groups was 2.3 × 10^8^, 1.5 × 10^8^, 1.2 × 10^8^, 5.4 × 10^7^, 1.6 × 10^7^, and 1.9 × 10^5^ CFU·mL^−1^, respectively. When ZIF-8 treated group showed slight effect on inhibiting MRSA infections, the number of MRSA in infected skin decreased significantly after treatment with low-dose or high-dose of ZIF-8-ICG under NIR laser irradiation. It was worth noting that only 30 min treatments of ZIF-8-ICG-L+NIR and ZIF-8-ICG-H+NIR can kill 93.1% and 99.9% MRSA efficiently, respectively (Figure 7f). Hence, all these results confirmed the outstanding in vivo activity of ZIF-8-ICG to eradicate multidrug-resistant bacterial infections.

**Figure 8:**
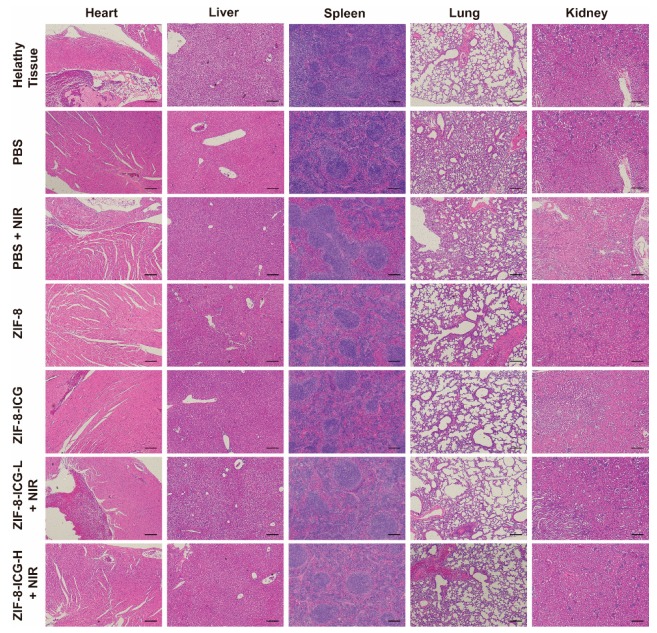
Hematoxylin and eosin-stained images of major organ sections after treatments with PBS, PBS+NIR, ZIF-8, ZIF-8-ICG, ZIF-8-ICG-L+NIR, and ZIF-8-ICG-H+NIR (Scale bar: 50 µm).

**Figure A2:**
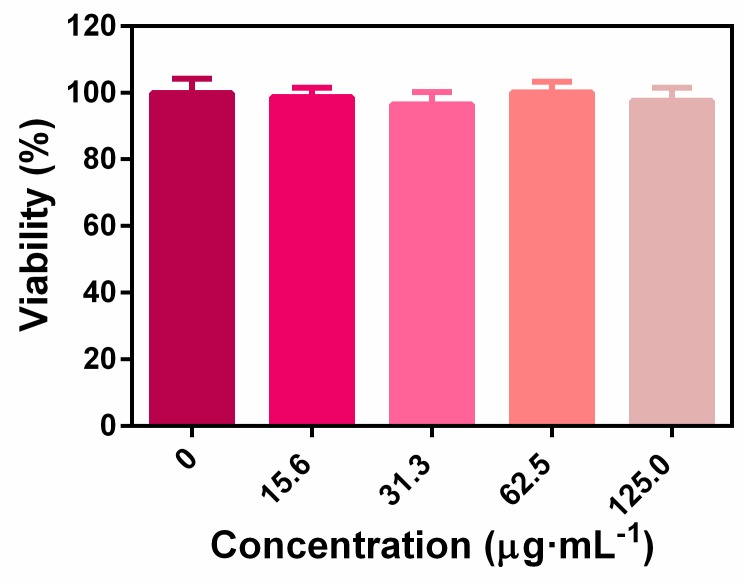
The 293T cell viabilities by incubating with various concentrations of ZIF-8-ICG.

The biocompatibility of nanoplatform was evaluated by in vitro cytotoxicity assay and in vivo animal experiments. As shown in Figure A2, the CCK-8 results revealed that the ZIF-8-ICG prepared in this research was cytocompatible. Subsequently, to further investigate whether the injection of nanoparticles into wound skin will induce cumulative toxicity to animal organs, the histological analyses of the heart, liver, spleen, lung, and kidney was performed on treated mice and healthy mice without bacterial infection [23]. As shown in Figure 8, the H&E stained sections of all samples were similar to the healthy group without apparent abnormities, such as swelling, ulceration, and erosion. Therefore, both in vitro and in vivo results illustrated that using ZIF-8-ICG for abscess healing is a kind of safe therapy.

## 4. Conclusions

In this study, a chemo-photothermal combinational system based on ZIF-8 and ICG was constructed for antibacterial therapy by a facile one-pot strategy. The biocompatible ZIF-8-ICG presented high ICG-loading capacity, outstanding photothermal conversion ability, and prominent in vitro antibacterial property. Moreover, the results of in vivo study further confirmed the remarkable bactericidal ability of ZIF-8-ICG under NIR irradiation. This novel chemo-photothermal combinational system was an alternative strategy for rapid and effective antibacterial therapy, which showed great potential in combating MDR bacteria.
