# Erratum: Wu, B.; et al. Metal–Organic Framework-Based Chemo-Photothermal Combinational System for Precise, Rapid, and Efficient Antibacterial Therapeutics. *Pharmaceutics* 2019, *11*, 463

**DOI:** 10.3390/pharmaceutics12020148

**Published:** 2020-02-12

**Authors:** Biyuan Wu, Jintao Fu, Yixian Zhou, Yin Shi, Jing Wang, Xiaoqian Feng, Yiting Zhao, Guiling Zhou, Chao Lu, Guilan Quan, Xin Pan, Chuanbin Wu

**Affiliations:** 1 School of Pharmaceutical Sciences, Sun Yat-sen University, Guangzhou 510006, China; wuby7@mail2.sysu.edu.cn (B.W.); fujt7@mail2.sysu.edu.cn (J.F.); zhouyx36@mail2.sysu.edu.cn (Y.Z.); shiy53@mail2.sysu.edu.cn (Y.S.); wangj527@mail2.sysu.edu.cn (J.W.); fengxq23@mail2.sysu.edu.cn (X.F.); zhaoyt8@mail2.sysu.edu.cn (Y.Z.); zhougling@mail2.sysu.edu.cn (G.Z.); panxin2@mail.sysu.edu.cn (X.P.); wuchuanb@mail.sysu.edu.cn (C.W.); 2 College of Pharmacy, Jinan University, Guangzhou 510632, China

The authors wish to make the following corrections to this paper [1]: the hematoxylin and eosin-stained images of kidney in the group of healthy tissue in Figure 8 of this work [1] inadvertently duplicated the kidney results of the PBS group. After the publication of this work, we noted the mistake and issued an erratum for correction. Figure 8 has now been corrected in this erratum.

The authors would like to apologize for any inconvenience caused to the readers by these changes.

## Figures and Tables

**Figure 8:**
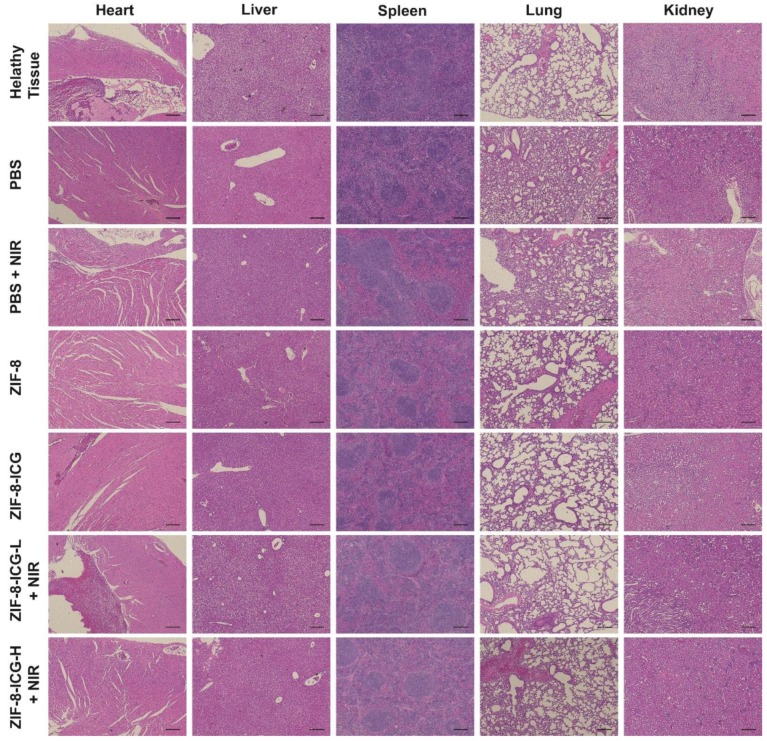
Hematoxylin and eosin-stained images of major organ sections after treatments with PBS, PBS+NIR, ZIF-8, ZIF-8-ICG, ZIF-8-ICG-L+NIR, and ZIF-8-ICG-H+NIR (Scale bar: 50 µm).
